# Supplementary material for: Spurious transcription causing innate immune responses is prevented by 5-hydroxymethylcytosine
Source: Nat Genet. 2022 Dec 20;55(1):100–11. doi: 10.1038/s41588-022-01252-3 (PMC9839451; doi:10.1038/s41588-022-01252-3)
Supplement: Supplementary file 1 — Supplementary Figs. 1–2. Contains information about FACS gating protocols. [file 41588_2022_1252_MOESM1_ESM.pdf]

nature genetics

Article https://doi.org/10.1038/s41588-022-01252-3

Spurious transcription causing innate immune responses is prevented by 5-hydroxymethylcytosine

In the format provided by the authors and unedited

a

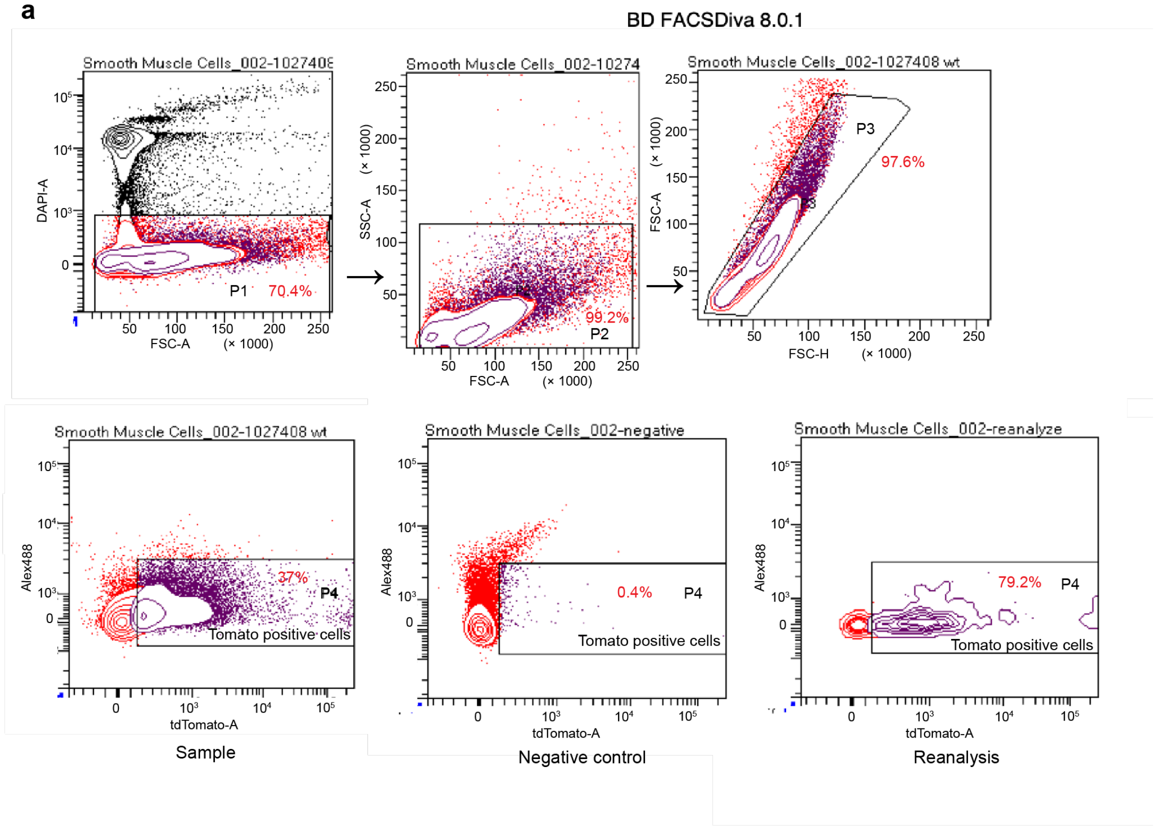

Supplementary Figure 1. Gating strategies for sorting of tdTomato+ smooth muscle cells. a, Cell sorting for the tdTomato+ population was achieved using a BD FACSAria III instrument and CD FACS Diva v8 software. The different areas for gating are indicated (parent percentages are shown in individual figures) (relates to Extended data Fig. 1n).

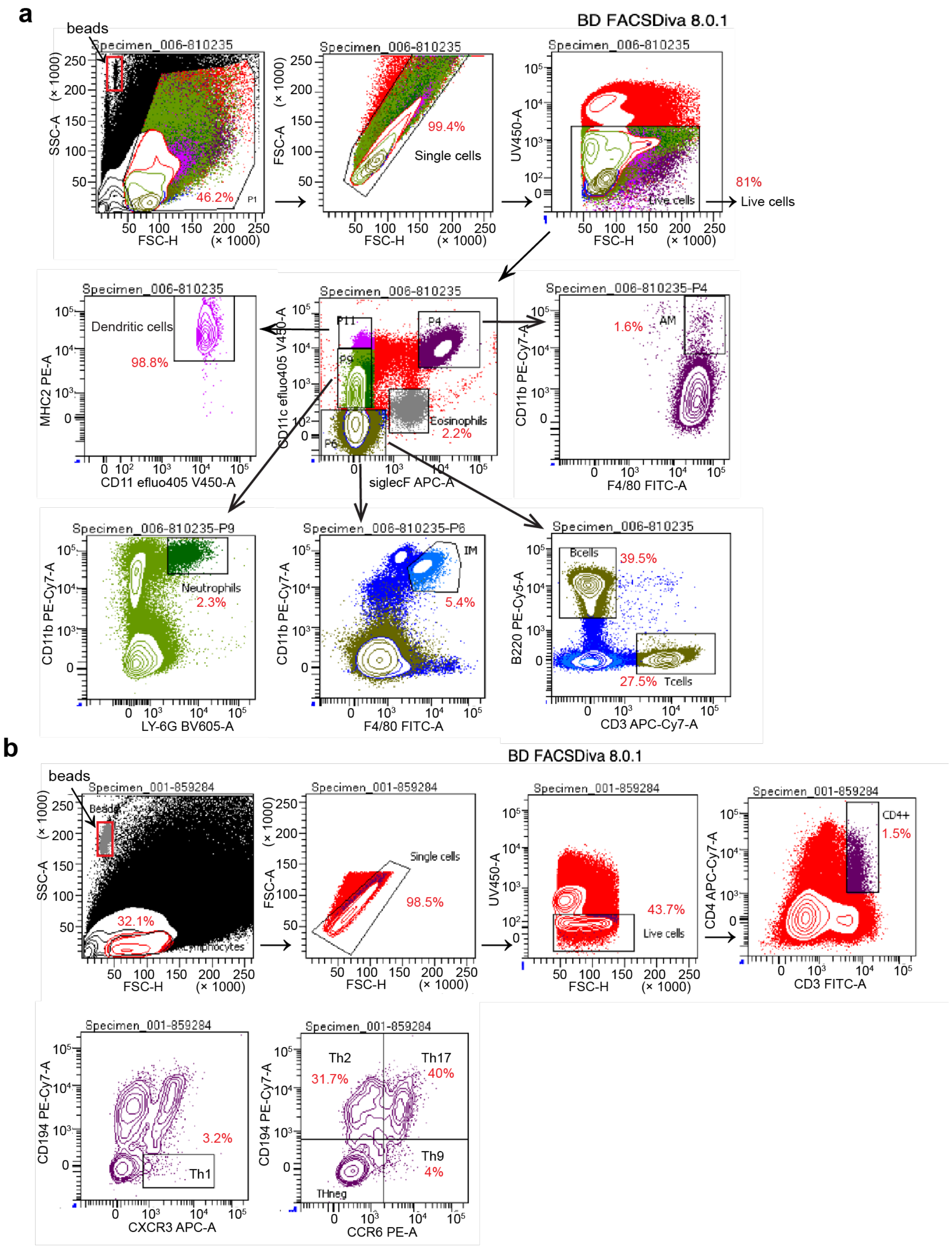

Supplementary Figure 2. Gating strategies for FACS analysis of inflammatory cells. a, Gating strategy for sorting of T cells, B cells, eosinophils, AMs, IMs, neutrophils, dendritic cells. Sorting for was accomplished using a BD FACSAria III instrument and CD FACS Diva v8 software. The different areas for gating are indicated. Parent percentages are shown in individual figures (relates to Fig. 6b, Extended data Fig. 8k). **b**, Gating strategy for sorting of Th1 cells, Th2 cells, Th9 cells, Th17 cells using a BD FACSAria III instrument and CD FACS Diva v8 software. Parent percentages are shown in individual figures (relates to Fig. 6d, Extended data Fig. 8l).
