# Supplementary material for: Spurious transcription causing innate immune responses is prevented by 5-hydroxymethylcytosine
Source: Nat Genet. 2022 Dec 20;55(1):100–11. doi: 10.1038/s41588-022-01252-3 (PMC9839451; doi:10.1038/s41588-022-01252-3)
Supplement: Supplementary file 2 — Reporting Summary [file 41588_2022_1252_MOESM2_ESM.pdf]

Corresponding author(s): Thomas Braun, Xuejun Yuan
Last updated by author(s): Oct 6, 2022

Reporting Summary

Nature Research wishes to improve the reproducibility of the work that we publish. This form provides structure for consistency and transparency in reporting. For further information on Nature Research policies, see our Editorial Policies and the Editorial Policy Checklist.

Statistics

For all statistical analyses, confirm that the following items are present in the figure legend, table legend, main text, or Methods section.

n/a	Confirmed	
☐	☒	The exact sample size (*n*) for each experimental group/condition, given as a discrete number and unit of measurement
☐	☒	A statement on whether measurements were taken from distinct samples or whether the same sample was measured repeatedly
☐	☒	The statistical test(s) used AND whether they are one- or two-sided<br>*Only common tests should be described solely by name; describe more complex techniques in the Methods section.*
☐	☒	A description of all covariates tested
☐	☒	A description of any assumptions or corrections, such as tests of normality and adjustment for multiple comparisons
☐	☒	A full description of the statistical parameters including central tendency (e.g. means) or other basic estimates (e.g. regression coefficient) AND variation (e.g. standard deviation) or associated estimates of uncertainty (e.g. confidence intervals)
☐	☒	For null hypothesis testing, the test statistic (e.g. *F*, *t*, *r*) with confidence intervals, effect sizes, degrees of freedom and *P* value noted<br>*Give P values as exact values whenever suitable.*
☒	☐	For Bayesian analysis, information on the choice of priors and Markov chain Monte Carlo settings
☒	☐	For hierarchical and complex designs, identification of the appropriate level for tests and full reporting of outcomes
☒	☐	Estimates of effect sizes (e.g. Cohen's *d*, Pearson's *r*), indicating how they were calculated

Our web collection on statistics for biologists contains articles on many of the points above.

Software and code

Policy information about availability of computer code

Data collection

Zeiss Z1 Imager - software Zen2 6.1.7601
Leica SP8 - software LAS X 3.5.7.23225
FACS Aria TM III (BD Biosciences) - BD FACS Diva v8 Software
ChemiDoc™ MP Imaging System - Image Lab Version 6.1.0 Standard Edition
All the sequencing data were collected using Illumina Nextseq500 platform

Data analysis

GraphPad Prism 9
R 4.1.0
Macs2 peak caller version 2.1.0
IGV 2.3.52
BigWigAverageOverBed (UCSC Tools)
DESeq2 package from Bioconductor (https://bioconductor.org/packages/release/bioc/html/DESeq2.html)
GENCODE v25
FastQC tool http://www.bioinformatics.babraham.ac.uk/projects/fastqc.
Trimmomatic version 0.39, (http://www.usadellab.org/cms/?page=trimmomatic)
STAR 2.6.0c
Picard package http://broadinstitute.github.io/picard/, Broad Institute 2.17.10
MUSIC peak caller (https://github.com/gersteinlab/MUSIC)
UROPA (https://github.com/loosolab/UROPA) using the GENCODE annotation (V.16).
BWA software (v0.5.9)
HOMER

For manuscripts utilizing custom algorithms or software that are central to the research but not yet described in published literature, software must be made available to editors and reviewers. We strongly encourage code deposition in a community repository (e.g. GitHub). See the Nature Research guidelines for submitting code & software for further information.

Data

Policy information about availability of data

All manuscripts must include a data availability statement. This statement should provide the following information, where applicable:

- Accession codes, unique identifiers, or web links for publicly available datasets
- A list of figures that have associated raw data
- A description of any restrictions on data availability

GEO accession number: GSE166816. Figure 2a, Figure 2e, Figure 4a, b, e, g, Extended Data Figure 4a, b, Extended Data Figure 5a, b, are associated with this data.
GEO accession number: GSE 166815. Figure 2d, e, f, Figure 4a, Extended data Figure 3b, c, Extended data Figure 4d, are associated with this data.
GEO accession number: GSE 168206. Figure 4a, c, d, e, f, g, Extended data Figure 4c, d, are associated with this data.
Accession number: E-MTAB-10144 at www.ebi.ac.uk/arrayexpress/ (Password: dwmfpwxk). Extended data Figure 6d is associated with this data.
GEO accession number (Nano-seal): GSE 202201. Figure 4g, Extended data Figure 3b, c, Extended data Figure 5b are associated with this data.
GEO accession number (H3K36me3 ChIPseq): GSE 201924. Figure 3d, e, Extended data Figure 3h are associated with this data.
All data were already released to the public.

Field-specific reporting

Please select the one below that is the best fit for your research. If you are not sure, read the appropriate sections before making your selection.

☒ Life sciences ☐ Behavioural & social sciences ☐ Ecological, evolutionary & environmental sciences

For a reference copy of the document with all sections, see nature.com/documents/nr-reporting-summary-flat.pdf

Life sciences study design

All studies must disclose on these points even when the disclosure is negative.

Sample size

Sample size were determined based on established practice and applicable standards. We opted for sample sizes which are commonly used sample size in the field. For in vivo studies, a minimum of three biological replicates were analyzed. For In vitro studies, experiments in which data were not quantified were performed with at least two replicates. Each experiment in which data were quantified was performed with at least 3 replicates.

Data exclusions

No data were excluded.

Replication

All in vivo studies were performed once with indicated number of animals. The in vitro study were performed at least 3 times independently and each replicate was successful. Sample sizes, statistical analyses and significance levels are all indicated in the figure legends or the method part.

Randomization

All animals were numbered and experiments were performed in a blinded pattern. After data collection, genotypes were revealed and animals assigned to groups based on genotype for data analysis. For in vitro study, the cultured cell plates were randomly assigned to each group for respective treatment.

Blinding

For in vivo study, all animals were numbered and experiments were performed in a blinded pattern. After data collection, genotypes were revealed and animals assigned to groups based on genotype for data analysis. In vitro experiments were not blinded during data collection or analysis since we know the treatment of each group before data collection. Positive controls, negative controls and samples were analyzed in exactly the same manner.

Reporting for specific materials, systems and methods

We require information from authors about some types of materials, experimental systems and methods used in many studies. Here, indicate whether each material, system or method listed is relevant to your study. If you are not sure if a list item applies to your research, read the appropriate section before selecting a response.

Materials & experimental systems

n/a	Involved in the study
	☒ Antibodies
	☒ Eukaryotic cell lines
☒	☐ Palaeontology and archaeology
	☒ Animals and other organisms
	☒ Human research participants
☒	☐ Clinical data
☒	☐ Dual use research of concern

Methods

n/a	Involved in the study
	☒ ChIP-seq
	☒ Flow cytometry
☒	☐ MRI-based neuroimaging

Antibodies

Antibodies used

5hmC (1:5000, Active motif, #39769), 5mC (1:5000, Eurogentec, #81103), 5fC (1:5000, Active motif, #61223), 5caC (1:5000, Active motif, #61225), TET3 (1:1000, GeneTex, #GTX121453), TET3 (1:500, Active motif, #61395), TET2 (1:1000, BETHYL, #A303-604A), RNA Pol II Pan (1:1000, Active motif, #39097), RNA Pol II ser2 (1:1000, Abcam, #ab5095), RNA Pol II ser5 (1:1000, Abcam, #ab5408), SETD2 (Abclonal, #A3194), NSD3 (1:1000, Proteintech, #11345-1-AP), H3K36me3 (1:1000, Abcam, #ab9050), TLR7 (1:1000, Proteintech, #17232-1-AP), Myd88 (1:1000, Santa Cruz, #sc-74532), EEA1 (1:1000, Cell signaling, #3288T), RAB7 (1:1000, Cell signaling, #9367T), alpha-SMA-FITC (1:2000, Sigma, #F3777), alpha-SMA-Cy3 (1:2000, Sigma, #C6198), CCSP (1:1000, Millipore, #07-623), alpha-Tubulin (1:1000, Sigma, #T6074), Mucin-5AC (1:1000, Abcam, #ab3649), Ki67 (1:1000, Abcam, #ab92742), AGR2 (1:1000, Abcam, #ab76473), CD68 (1:1000, eBioscience, #E24592), Collagen I (1:1000, Proteintec, #14695-1-AP), alpha-SMA (1:1000, Sigma, #A2547), MYH11 (1:1000, Abcam, #ab5319), TAGLN (1:1000, Proteintec, #10493-1-AP), Vimentin (1:1000, Abcam, #ab92547), TPM4 (1:1000, Millipore, #AB5449), Pan actin (1:1000, Cell signaling, #4968), Histone H3 (1:5000, Abcam, #ab1791), CD3 (BD, #560590), CD45R (1:1000, Invitrogen, #4329548), Siglec-F (1:100, Miltenyi Biotec, #5161104265), CD11C (1:100, eBioscience, #4300054), CD11B (1:100, eBioscience, #4289817), F4/80, (1:100, Bio-Rad, #MCA497FT), LY6G (1:100, BD, #563005), MHCII (1:100, eBioscience, #4271684), CD4 (1:100, BD, #561830), CD3 (1:100, BD, #561798), CD196 (1:100, Biolegend, #129803), CD194 (1:100, Biolegend, #131213), CD183 (1:100, Biolegend, #126511).

Validation

5hmC (1:5000, Active motif, #39769), validated by dot blot with 27 base oligonucleotide containing 5-hydroxymethylcytosine (1.2 ng); validated by Methyl DNA immunoprecipitation with 25ng DNA, this antibody was validated with mouse genomic DNA for Dot blot from publications, such as Shu,L and et al, BMC genomic, 2016. 5mC (1:5000, Eurogentec, #81103), validated by Ito et al., 2010, Nature. 5fC (1:5000, Active motif, #61223), validated by dot blot with oligo containing 5-formylcytidine. This antibody has been validated with mouse tissue by previous publications, such as Fang, S, et al, Nat Common, 2019. 5caC (1:5000, Active motif, #61225), validated by dot blot with oligo containing 5-carboxylcytidine. This antibody has been validated with mouse tissue by previous publications, such as Fang, S, et al, Nat Common, 2019. TET3 (1:1000, GeneTex, #GTX121453), validated by immunoprecipitates TET3 protein with TET3 gene transfected 293T cells; validated by immunofluorescent analysis by HeLa cells. TET2 (1:1000, BETHYL, #A303-604A), validated by immunofluorescent analysis by human ovarian carcinoma sections. RNA Pol II Pan (1:1000, Active motif, #39097), validated by western blot analysis by HeLa nuclear extracts. RNA Pol II ser2 (1:1000, Abcam, #ab5095), validated by western blot with HeLa whole cell lysate. RNA Pol II ser5 (1:1000, Abcam, #ab5408), validated by chromatin immunoprecipitation with U2OC cells. SETD2 (Abclonal, #A3194), validated by western blot with mouse brain, mouse kidney and mouse liver. NSD3 (1:1000, Proteintech, #11345-1-AP), validated by immunofluorescent analysis by HeLa cells. H3K36me3 (1:1000, Abcam, #ab9050), validated by chromatin immunoprecipitation with U2OS cells. TLR7 (1:1000, Proteintech, #17232-1-AP), validated by immunohistochemical analysis with paraffin-embedded human small intestine tissue slide. MYD88 (1:1000, Santa Cruz, #sc-74532), validated by immunohistochemical analysis with paraffin-embedded human small intestine tissue. EEA1 (1:1000, Cell signaling, #3288T), validated by western blot with HeLa cell extracts. RAB7 (1:1000, Cell signaling, #9367T), validated by western blot with HeLa cell extracts. alpha-SMA-FITC (1:2000, Sigma, #F3777), validated by immunohistochemical analysis with paraffin-embedded tissue section of human appendix. This antibody has been validated with mouse tissue with previous publications, such as Dai, Z and et al, Circulation, 2016.alpha-SMA-Cy3 (1:2000, Sigma, #C6198), validated by immunohistochemical analysis with paraffin-embedded tissue section of human appendix. CCSP (1:1000, Millipore, #07-623), validated by immunohistochemical analysis with paraffin-embedded murine lung section. alpha-Tubulin (1:1000, Sigma, #T6074), validated by immunofluorescent analysis by CFB cells. Mucin-5AC (1:1000, Abcam, #ab3649), validated by immunohistochemical analysis with human gastric carcinoma tissue. Ki67 (1:1000, Abcam, #ab92742), validated by immunohistochemical analysis with human colorectal adenocarcinoma cell line. AGR2 (1:1000, Abcam, #ab76473), validated by immunofluorescent analysis with human breast adenocarcinoma epithelial cell. CD68 (1:1000, eBioscience, #E24592), validated by immunofluorescence analysis with THP-1 cells differentiated into macrophage. Collagen I (1:1000, Proteintec, #14695-1-AP), validated by immunohistochemical analysis with paraffin-embedded human skin cancer tissue slide. alpha-SMA (1:1000, Sigma, #A2547), validated by western blot with C2C12 whole cell lysate. MYH11 (1:1000, Abcam, #ab5319), validated by immunohistochemical analysis with frozen mouse small intestine tissue section. TAGLN (1:1000, Proteintec, #10493-1-AP), validated by immunofluorescent analysis with fixed mouse heart tissue. Vimentin (1:1000, Abcam, #ab92547), validated by immunohistochemical analysis with paraffin embedded mouse kidney. TPM4 (1:1000, Millipore, #AB5449), validated by Kramann et al., 2016, Cell Stem Cell. Pan actin (1:1000, Cell signaling, #4968), validated by western blot with HeLa whole cell lysate. Histone H3 (1:5000, Abcam, #ab1791), validated by western blot with HEK293 whole cell lysate. CD3 (BD, #560590), validated by flow cytometric analysis with mouse splenocytes. CD45R (1:1000, Invitrogen, #4329548), validated by flow cytometric analysis with mouse splenocytes. Siglec-F (1:100, Miltenyi Biotec, #5161104265), validated by flow cytometry analysis with bone marrow cells. CD11c (1:100, eBioscience, #4300054), validated by flow cytometric analysis with mouse splenocytes. CD11b (1:100, eBioscience, #4289817), validated by flow cytometric analysis with mouse bone marrow cells. F4/80, (1:100, Bio-Rad, #MCA497FT), validated by flow cytometric analysis with mouse peritoneal macrophages. LY6G (1:100, BD, #563005), validated by flow cytometric analysis with mouse bone marrow leukocytes. MHCII (1:100, eBioscience, #4271684), validated by flow cytometric analysis with mouse splenocytes. CD4 (1:100, BD, #561830), validated by flow cytometric analysis with mouse splenocytes. CD3 (1:100, BD, #561798),

validated by flow cytometric analysis with mouse splenocytes. CD196 (1:100, Biolegend, #129803), validated by flow cytometric analysis with mouse splenocytes. CD194 (1:100, Biolegend, #131213), validated by He W et al., 2016, Immunity. CD183 (1:100, Biolegend, #126511), validated by flow cytometric analysis with mouse splenocytes.

Eukaryotic cell lines

Policy information about cell lines

Cell line source(s)	HEK293T, HEK293, HeLa and MLE12 cells were purchased from ATCC.
Authentication	All human cell lines were obtained from ATCC and maintained as instructed. The cell was checked for morphology for cell authentication.
Mycoplasma contamination	tested negative for mycoplamsa
Commonly misidentified lines (See ICLAC register)	No commonly misidentified cell lines were used

Animals and other organisms

Policy information about studies involving animals; ARRIVE guidelines recommended for reporting animal research

Laboratory animals	All mice were maintained on a C57BL/6 background, and littermates were used as controls in all experiments. All experiments were performed on balanced cohorts of male and female mice as our initial data did not indicate significant differences in lung airway morphological changes between females and males. ROSA26tdTomato mice were obtained from The Jackson Laboratory. To facilitate the lineage tracing experiment for IF and FACS sorting, we crossed the ROSA26tdTomasto with Tet3 flox mice to label smooth muscle cell specifically. The Tamoxifen injection was performed when the animals were 8 weeks old.
Wild animals	Studies did not involve wild animals.
Field-collected samples	Studies did not involve samples collected in the field.
Ethics oversight	All animal experiments were done in accordance with the Guide for the Care and Use of Laboratory Animals published by the US National Institutes of Health (NIH Publication No. 85-23, revised 1996) and were approved by the responsible Committee for Animal Rights Protection of the State of Hessen (Regierungspraesidium Darmstadt, Wilhelminenstr. 1-3, 64283 Darmstadt, Germany) with the project number B2/1125, B2/1137 and B2/1056.

Note that full information on the approval of the study protocol must also be provided in the manuscript.

Human research participants

Policy information about studies involving human research participants

Population characteristics	Human donor samples, human COPD samples, human cystic fibrosis samples were provided by the DZL Biobank (Deutsches Zentrum für Lungenforschung).
Recruitment	Human samples were obtained during lung transplantation of human COPD and cystic fibrosis patients. Donor lung material was obtained as a result of atypical resections undertaken to adjust the donor organ to the recipient's thoracal cavity.
Ethics oversight	The study protocol for tissue donation was approved by the Ethics Committee of the Department of Human Medicine of Justus Liebig University Hospital, Giessen, Germany, in accordance with national law and with the 'Good Clinical Practice/ International Conference on Harmonization' guidelines. Approval code: (Az. 58/15 and 111/08).<br>Human asthma samples were obtained from the BioMaterialBank Nord, Clinical and Experimental Pathology Medicine, Research Center Borstel. Approval to use human samples for research was granted by the Ethics Committee of the University of Lübeck (Az 12-220 and 14-225).

Note that full information on the approval of the study protocol must also be provided in the manuscript.

ChIP-seq

Data deposition

☒ Confirm that both raw and final processed data have been deposited in a public database such as GEO.

☒ Confirm that you have deposited or provided access to graph files (e.g. BED files) for the called peaks.

Data access links<br>*May remain private before publication.*	https://www.ncbi.nlm.nih.gov/geo/query/acc.cgi?acc=GSE166815
Files in database submission	Pol II ser5 ChIPseq:<br>GSM5086002 SMCs, ChIP, Mut_IP_1<br>GSM5086003 SMCs, ChIP, Mut_IP_2<br>GSM5086004 SMCs, ChIP, Mut_Input_1

GSM5086005 SMCs, ChIP, Mut_Input_2
GSM5086006 SMCs, ChIP, WT_IP_1
GSM5086007 SMCs, ChIP, WT_IP_2
GSM5086008 SMCs, ChIP, WT_Input_1
GSM5086009 SMCs, ChIP, WT_Input_2

H3K36me3 ChIPseq:
GSM6081162 H3K36me3 ChIPSeq [Mut_2]
GSM6081163 H3K36me3 ChIPSeq [Mut_3]
GSM6081164 Input DNA [Mut_2]
GSM6081165 Input DNA [Mut_3]
GSM6081166 H3K36me3 ChIPSeq [WT_2]
GSM6081167 H3K36me3 ChIPSeq [WT_3]
GSM6081168 Input DNA [WT_2]
GSM6081169 Input DNA [WT_3]

Genome browser session (e.g. UCSC)

N/A

Methodology

Replicates

Two biological replicates for RNA Pol II pSer5 ChIP-seq and H3K36me3 ChIP-seq.

Sequencing depth

Pol II ser5 ChIPseq:
Single end.
Length: 75bp
WT1 21M 69% aligned
WT2 23M 72% aligned
MUT1 16M 68% aligned
MUT2 24M 73% aligned

H3K36me3 ChIPseq:
WT2 21M 95% aligned
WT3 23M 94% aligned
MUT2 16M 94% aligned
MUT3 24M 94% aligned

Antibodies

RNA Pol II ser5 (Abcam, #ab5408), H3K36me3 (Abcam, #ab9050)

Peak calling parameters

Peaks were annotated with the promoter (TSS +- 5000 nt) of genes closely located to the centre of the peak based on reference data from GENCODE v25. The degree of reproducibility between samples was assessed by Spearman correlations. To permit comparative display of samples in IGV, raw BAM files were scaled with DESeq2 size factors based on all unified peaks using bedtools genomecov resulting in normalized BigWig files. Finally, DESeq2 was used to identify significantly differentially modified peaks based on background-corrected read counts from recounted unified peak regions.

Data quality

Pol II ser5 ChIPseq: Reproducible peaks in WT1 and WT2: 2949; promoter intersected peaks: 2325
Reproducible peaks in MUT1 and MUT2: 2935; promoter intersected peaks: 2312
H3K36me3 ChIPseq: Reproducible peaks in WT2 and WT3: 15886;
Reproducible peaks in MUT2 and MUT3: 15835

Software

Macs2 peak caller version 2.1.0
IGV 2.3.52
BigWigAverageOverBed (UCSC Tools)
DESeq2 package from Bioconductor
GENCODE v25
FastQC tool http://www.bioinformatics.babraham.ac.uk/projects/fastqc.

Flow Cytometry

Plots

Confirm that:

☒ The axis labels state the marker and fluorochrome used (e.g. CD4-FITC).

☒ The axis scales are clearly visible. Include numbers along axes only for bottom left plot of group (a 'group' is an analysis of identical markers).

☒ All plots are contour plots with outliers or pseudocolor plots.

☒ A numerical value for number of cells or percentage (with statistics) is provided.

Methodology

Sample preparation

After sacrificing experimental mice, blood was removed by perfusion with cold PBS through the right ventricle prior to lung dissection. Lung tissues were dissected and minced into small pieces before incubation in 3 ml digestion buffer (DPBS containing Collagenase type 2 (2mg/ml, Worthington), Elastase (0.04mg/ml, Worthington) and DNase (5U/ml, Roche) with frequent agitation at 37o C for 10min. Immediately afterwards, 10-times the volume of cold DMEM supplemented with 10% FBS was added to single-cell suspensions. Cells were mechanically dissociated by passing 4-5 times through a 30 ml syringe and consecutive filtering through 100-, 70- and 40-µm cell strainers (BD Biosciences). The filtrate was centrifuged at 300 g, room temperature (RT) for 10min. Pellets were re-suspended in 1ml pre-cooled MACS buffer (Cat# A9576, Miltenyi Biotec) with 1% BSA. After 5min centrifugation at 300g, cell pellets were re-suspended in 90µL MACS buffer and incubated with 10 µL CD45 MicroBeads (Cat#130-052-301) and anti-Ter-119 MicroBeads (Cat#130-049-901) at 4o C for 15min to remove hematopoietic cells. After washing with MACS buffer, cells were loaded into pre-conditioned LS columns (Miltenyi Biotec) on a MACS separator and the flow-through containing unlabeled cells was collected.

Instrument

FACS sorting for smooth muscle cells: FACSAriaTM III (BD Biosciences)
FACS analysis for lung inflammatory cells: LSR Fortessa (BD Biosciences)

Software

BD FACS Diva v8 software

Cell population abundance

Sorted cells were reanalyzed to assess purity. A 80% purity was achieved. Data related to reanalysis of sorted cells is shown in Supplementary Figure 1.

Gating strategy

The gating strategy is shown in Extended data Fig. 1j, Supplementary Figure 1, Supplementary Figure 2.

☒ Tick this box to confirm that a figure exemplifying the gating strategy is provided in the Supplementary Information.
