# Supplementary figures and images for: Spurious transcription causing innate immune responses is prevented by 5-hydroxymethylcytosine
Source: Nat Genet. 2022 Dec 20;55(1):100–11. doi: 10.1038/s41588-022-01252-3 (PMC9839451; doi:10.1038/s41588-022-01252-3)

Fig. 1d

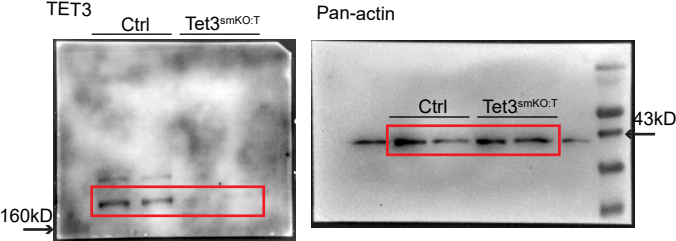

Supplement: Source Data Fig. 1 — Unprocessed blots or gels. [file 41588_2022_1252_MOESM6_ESM.pdf]

Fig. 3a

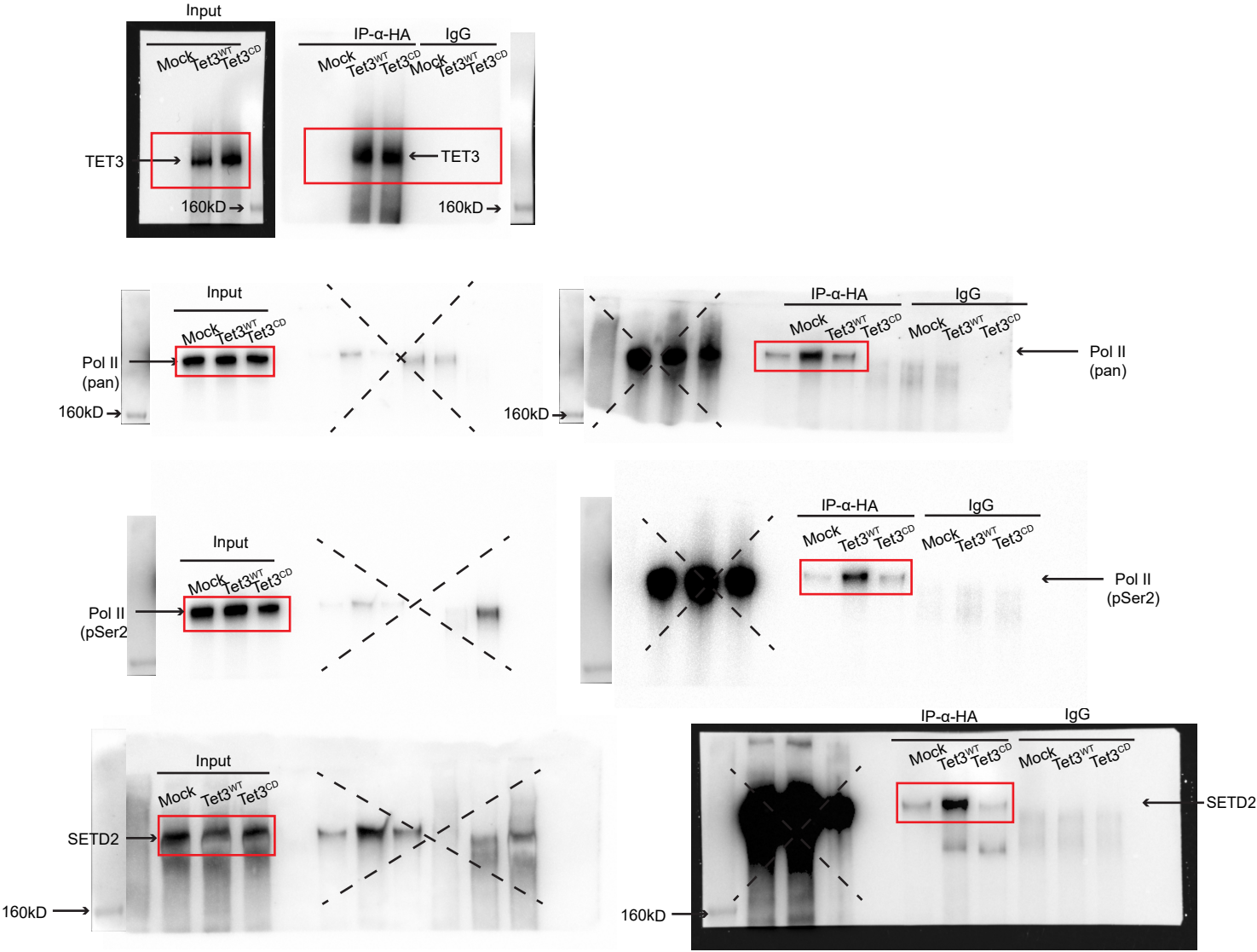

Fig. 3c

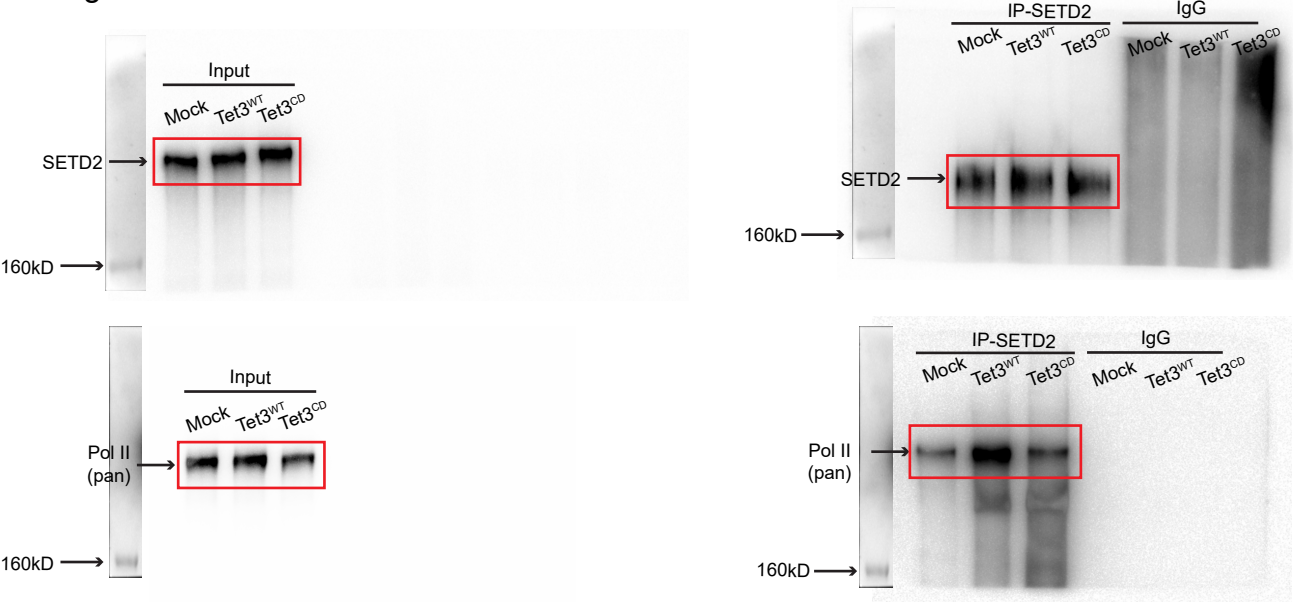

Supplement: Source Data Fig. 3 — Unprocessed blots or gels. [file 41588_2022_1252_MOESM9_ESM.pdf]

Fig. 7b

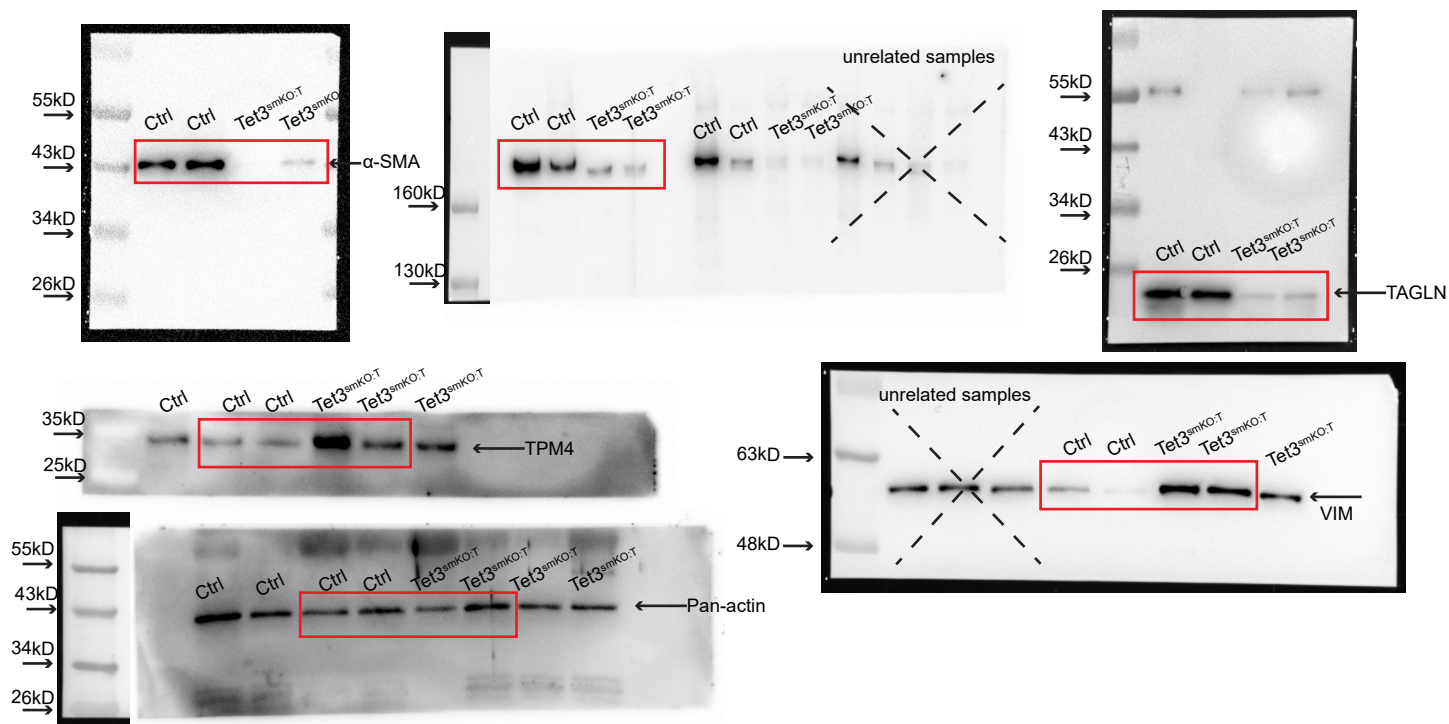

Fig. 7c

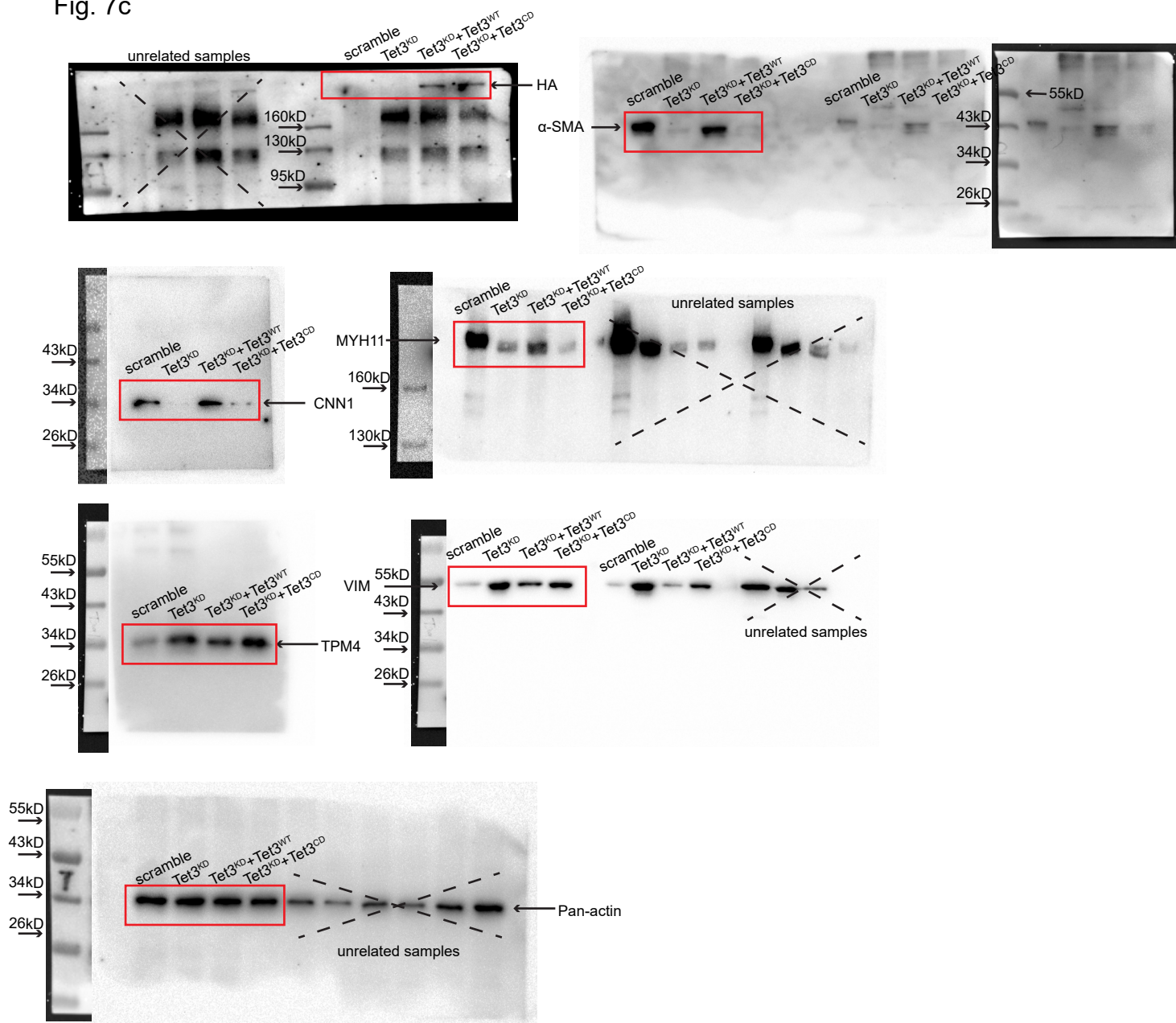

Fig. 7f

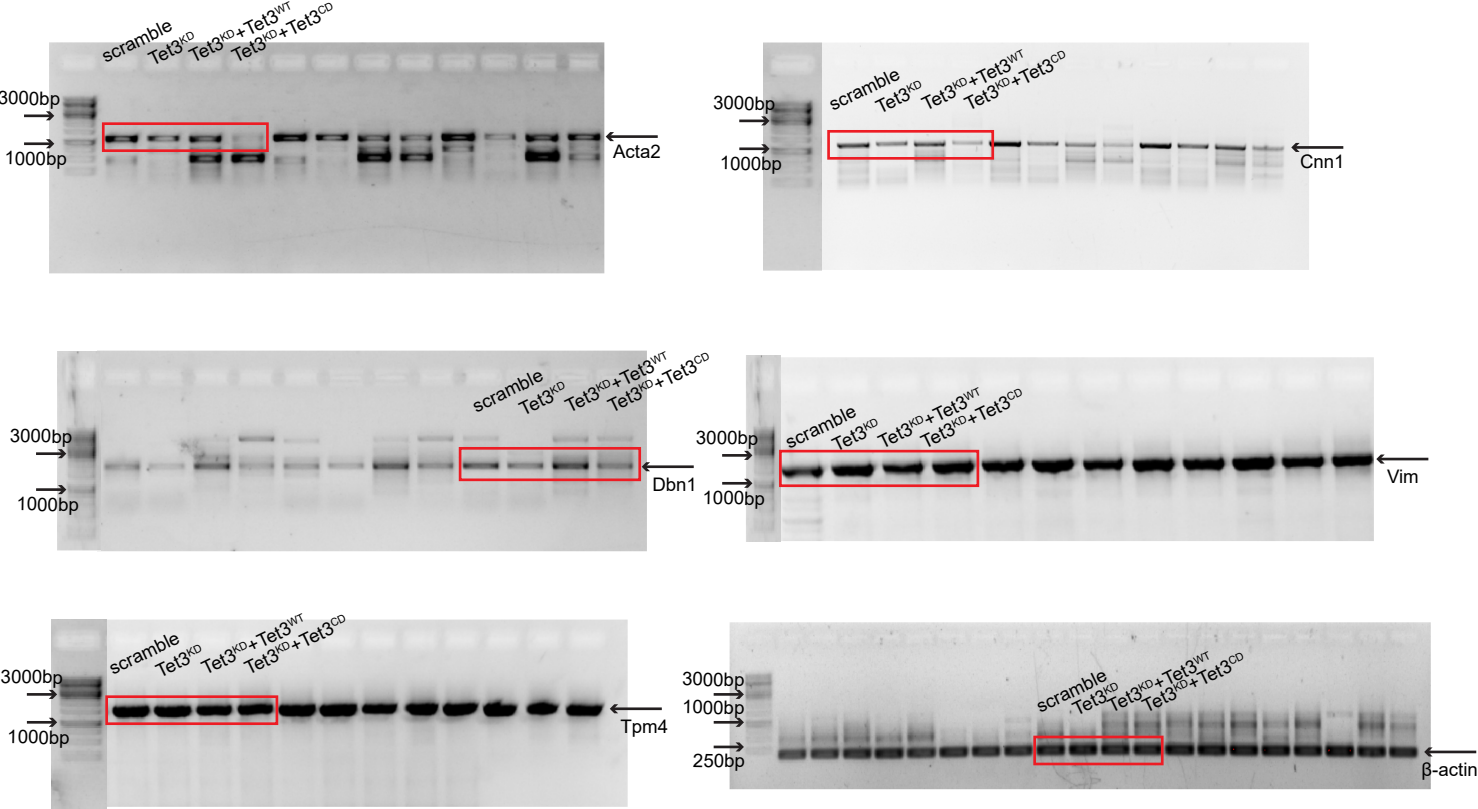

Supplement: Source Data Fig. 7 — Unprocessed blots or gels. [file 41588_2022_1252_MOESM14_ESM.pdf]

Extended data Fig. 1o

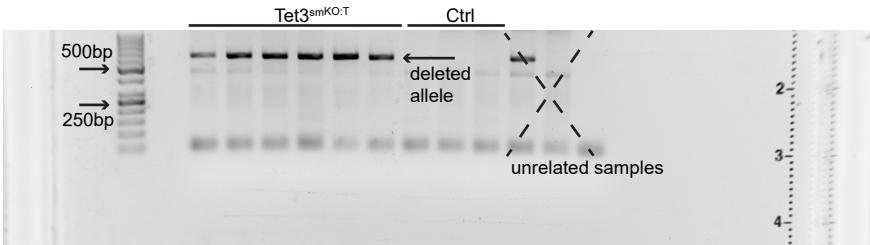

Supplement: Extended Data Fig. 1 — Unprocessed blots or gels. [file 41588_2022_1252_MOESM17_ESM.pdf]

Extended data Fig. 2b

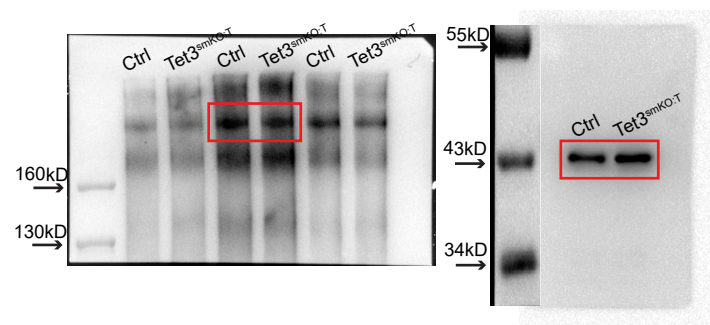

Extended data Fig. 2h

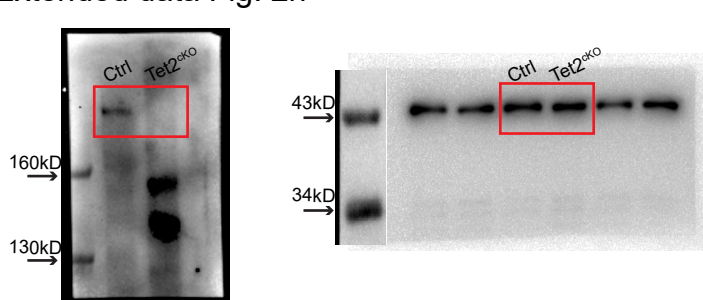

Extended data Fig. 2d

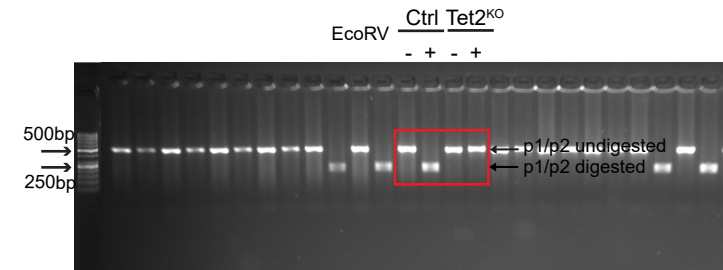

Supplement: Extended Data Fig. 2 — Unprocessed blots or gels. [file 41588_2022_1252_MOESM19_ESM.pdf]

Extended data Fig. 3e

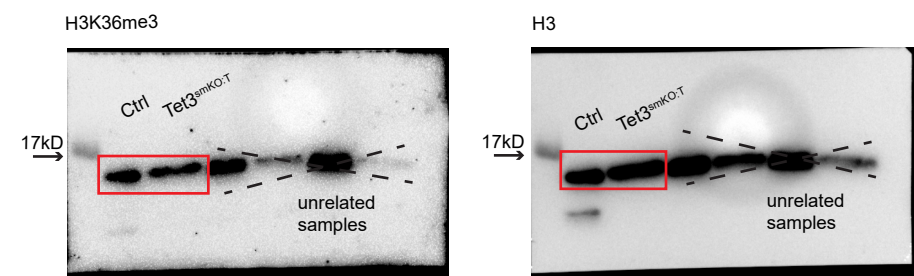

Supplement: Extended Data Fig. 3 — Unprocessed blots or gels. [file 41588_2022_1252_MOESM21_ESM.pdf]

Extended data Fig. 4j

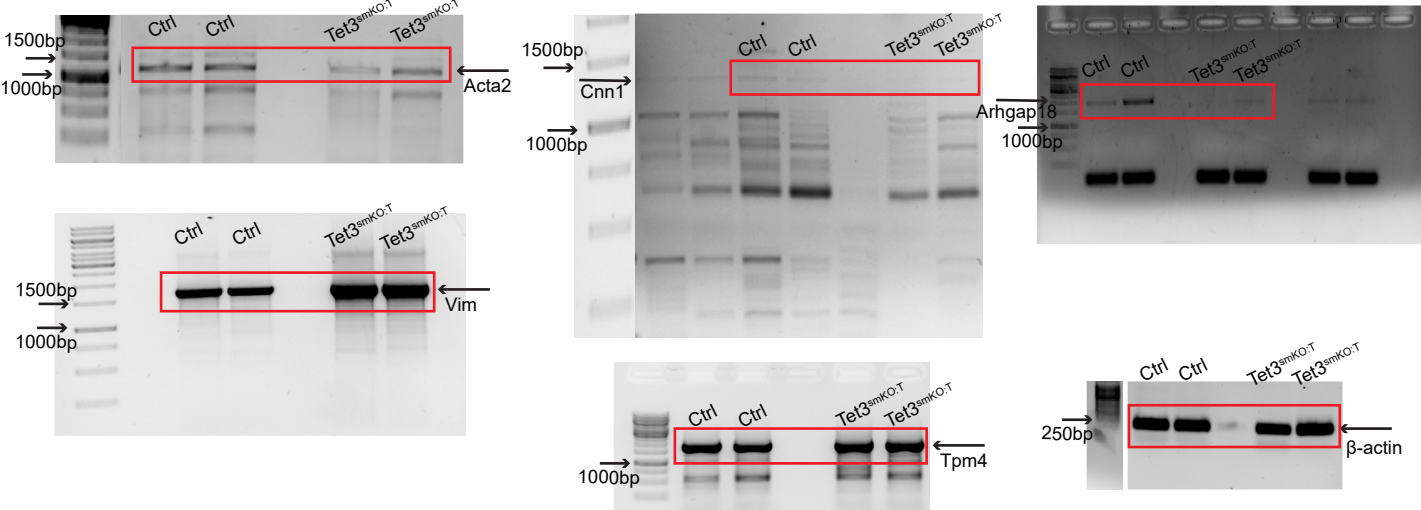

Supplement: Extended Data Fig. 4 — Unprocessed blots or gels. [file 41588_2022_1252_MOESM23_ESM.pdf]

Extended data Fig. 6b

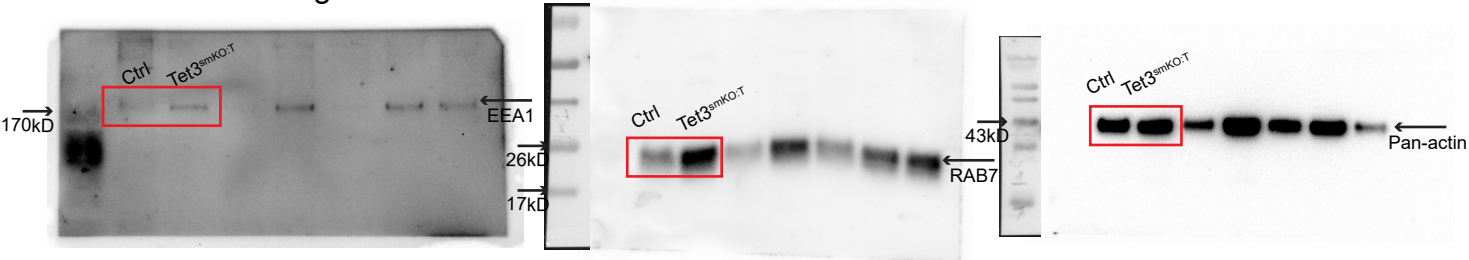

Extended data Fig. 6g

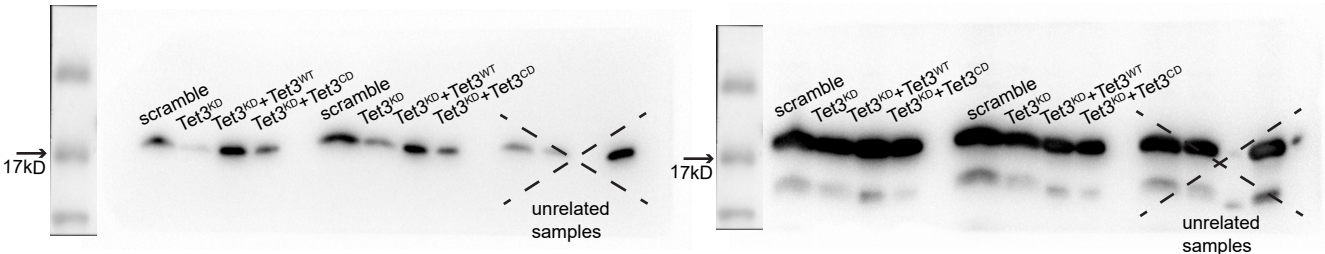

Supplement: Extended Data Fig. 6 — Unprocessed blots or gels. [file 41588_2022_1252_MOESM26_ESM.pdf]
